# Assessment of the performance of the O-RADS MRI score for the evaluation of adnexal masses, with technical notes

**DOI:** 10.1590/0100-3984.2021.0050

**Published:** 2022

**Authors:** Patrick Nunes Pereira, Adriana Yoshida, Luís Otavio Sarian, Ricardo Hoelz de Oliveira Barros, Rodrigo Menezes Jales, Sophie Derchain

**Affiliations:** 1 Department of Obstetrics and Gynecology, Faculdade de Ciências Médicas da Universidade Estadual de Campinas (FCM-Unicamp), Campinas, SP, Brazil.; 2 Department of Radiology, Hospital das Clínicas da Faculdade de Ciências Médicas da Universidade Estadual de Campinas (FCM-Unicamp), Campinas, SP, Brazil.

**Keywords:** Magnetic resonance imaging, Adnexal diseases/diagnostic imaging, Ovarian neoplasms/diagnostic imaging, Ressonância magnética, Doenças dos anexos/diagnóstico por imagem, Neoplasias ovarianas/diagnóstico por imagem

## Abstract

**Objective:**

To assess the performance of the Ovarian-Adnexal Reporting and Data System Magnetic Resonance Imaging (O-RADS MRI) score in the evaluation of adnexal masses and to provide technical notes about its current MRI parameters and concepts.

**Materials and Methods:**

This was a prospective study of 226 patients with 287 adnexal masses (190 submitted to surgery or biopsy and 97 followed for at least one year). We calculated the sensitivity, specificity, positive predictive value, and negative predictive value for the O-RADS MRI score, using ≥ 4 as the cutoff for malignancy. We performed a technical analysis of the main updates to the score, announced in September 2020 by the American College of Radiology, in comparison with the original (2013) version.

**Results:**

We found that an O-RADS MRI score of 4 or 5 was associated with malignancy of an adnexal mass, with a sensitivity of 91.11% (95% CI: 83.23-96.08), specificity of 94.92% (95% CI: 90.86-97.54), positive predictive value of 89.13% (95% CI: 81.71-93.77), negative predictive value of 95.90% (95% CI: 92.34-97.84), and overall accuracy of 93.73% (95% CI: 90.27-96.24).

**Conclusion:**

Our findings support the use of the O-RADS MRI score for evaluating adnexal masses, especially those considered indeterminate on ultrasound. The updates made recently to the O-RADS MRI score facilitate its interpretation and will allow its more widespread use, with no loss of diagnostic accuracy.

## Abstract (pt)

**Objetivo:**

Determinar o desempenho do escore de ressonância magnética para lesões anexiais ovarianas (escore O-RADS RM), com notas técnicas sobre seus atuais parâmetros e conceitos de RM utilizados.

**Materiais e Métodos:**

Este estudo incluiu 226 pacientes com 287 massas anexiais (190 pacientes submetidas a cirurgia/biópsia e 97 pacientes com pelo menos um ano de seguimento). Calculamos sensibilidade, especificidade, valores preditivos positivos e negativos para as categorias do escore O-RADS RM, usando ≥ 4 como ponto de corte para malignidade. Realizamos análise técnica das principais atualizações do escore, anunciadas em setembro de 2020 pelo American College of Radiology, em comparação com a versão original de 2013.

**Resultados:**

Escores O-RADS RM categorias 4 ou 5 foram associados com malignidade da massa anexial, com sensibilidade de 91,11% (IC 95%: 83,23-96,08), especificidade de 94,92% (IC 95%: 90,86-97,54), valor preditivo positivo de 89,13% (IC 95%: 81,71-93,77), valor preditivo negativo de 95,90% (IC 95%: 92,34-97,84) e acurácia de 93,73% (IC 95%: 90,27-96,24).

**Conclusão:**

Este estudo reforçou o uso do escore O-RADS RM para avaliar massas anexiais, principalmente as indeterminadas por ultrassom. As atualizações feitas recentemente no escore O-RADS RM facilitam sua interpretação e permitirão seu uso mais difundido, sem perder a precisão diagnóstica.

## INTRODUCTION

Adnexal masses are common findings in clinical practice^(1,2)^. The vast majority of adnexal masses are benign, malignant masses accounting for only a small proportion^(3,4)^. In 2020, the estimated number of cases of ovarian cancer worldwide was only 313,000^(5)^. Excluding malignancy of an adnexal mass through preoperative examinations is crucial for proper screening and treatment planning. It is recommended that a woman with a suspicious adnexal mass be referred to a surgeon specializing in gynecologic oncology^(6)^.

In most cases, screening for the risk of malignancy of an adnexal mass can be performed effectively by transvaginal ultrasound and the use of targeted algorithms, especially if the simple rules established by the International Ovarian Tumor Analysis (IOTA) group are applied^(7,8)^. However, approximately 20% of adnexal masses are considered indeterminate on ultrasound^(9,10)^.

Adnexal masses that are considered indeterminate represent a dilemma for the entire team that treats the affected patient, because such patients are at risk of unnecessary surgery. However, if the watchful waiting approach is adopted, the “window” of opportunity to diagnose cancer at an early stage may be missed. There is also a risk that the patient will be subjected to a surgical procedure performed by a non-specialist, if there is a false-negative test result or a lesion of non-ovarian origin^(6,11)^. According to the European Society of Urogenital Radiology^(12,13)^, magnetic resonance imaging (MRI) of the pelvis is indicated to assess an adnexal tumor that is considered indeterminate on transvaginal ultrasound.

In 2013, Thomassin-Naggara et al.^(14)^ presented a diagnostic algorithm for adnexal lesions that combines morphological features and functional MRI aspects to assign a numerical score. The system was initially called the ADNEX MR SCORING system and had five distinct categories (corresponding to scores from 1 to 5): categories 1, 2, and 3 were related to (probably) benign masses; and categories 4 and 5 were related to adnexal masses considered indeterminate or suspicious for malignancy. At cutoff scores of 4 and 5, the score had excellent accuracy (with a sensitivity of 93.5% and a specificity of 96.6%) for the detection of malignancy in an adnexal mass^(14)^. However, some technical aspects, such as the time-intensity of dynamic contrast-enhanced (DCE) studies of contrast-enhanced sequences (perfusion studies), which constituted one of the central elements of the ADNEX MR SCORING system, likely would have limited its use on a larger scale^(15)^. Studies have shown that it is possible to use less complex contrast-enhanced sequences, with no loss of accuracy^(16)^.

Recently, the ADNEX MR SCORING system was validated in a large prospective multicenter study conducted by the original authors^(17)^; some MRI aspects and parameters were improved, after which the score was adopted by the American College of Radiology, at which point it was renamed the Ovarian-Adnexal Reporting and Data System MRI (O-RADS MRI) score^(18,19)^. Some modifications were made; the new score incorporated the possibility of using lower temporal resolution in the DCE studies and even of performing a visual analysis of the enhancement pattern when a DCE study is not feasible^(18)^.

The O-RADS MRI score was designed to simplify and standardize the reporting of MRI of adnexal masses, in order to provide the clinician with the information necessary for the most appropriate management of patients, similar to the Breast Imaging Reporting and Data System, the result of which is linked to the clinical management of breast lesions^(20)^. In the O-RADS MRI score^(18)^, the absence of a suspicious adnexal lesion receives a score of 1; an adnexal lesion that is almost certainly benign receives a score of 2; a low-risk lesion receives a score of 3; an intermediate-risk lesion receives a score of 4; and a high-risk lesion receives a score of 5. In their subsequent study, Thomassin-Naggara et al.^(17)^ found that, for the detection of malignancy, a score of 4 or 5 had a sensitivity of 93.0% (95% CI: 89-96) and a specificity of 91.0% (95% CI: 89-93).

In this article, we evaluate the accuracy of the O-RADS MRI for evaluating adnexal masses in a large sample of lesions. We also provide technical notes on the updates in relation to the original score^(14)^.

## MATERIALS AND METHODS

This was a prospective study conducted at the Centro de Atenção Integral à Saúde da Mulher/Hospital da Mulher Prof. Dr. J. A. Pinotti, a tertiary cancer center operated by the Faculdade de Ciências Médicas da Universidade Estadual de Campinas (Unicamp), in the city of Campinas, SP, Brazil. The study was approved by the Unicamp Research Ethics Committee (Reference nos. 1092/2009 and 008/2010). All participating patients gave written informed consent.

**Figure 1:**
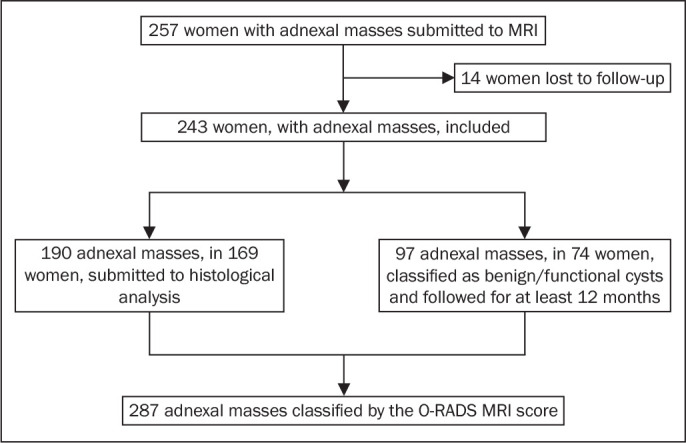
Flow chart of the patient selection process.

We randomly recruited women who were referred to our hospital for investigation of an adnexal mass between February 2014 and December 2020. To avoid any selection biases, we ensured that the recruiter had no knowledge of the clinical data (e.g., time of evolution), laboratory test results (serum levels of CA-125), or findings on imaging (pelvic ultrasound) for any given patient. An ultrasound evaluation of the pelvis was scheduled for each of the women enrolled. After ultrasound, 257 cases were scheduled for MRI, which was performed at the Hospital Estadual Sumaré, in the city of Sumaré, SP, Brazil, a Unicamp-affiliated hospital located near the Hospital da Mulher Prof. Dr. J. A. Pinotti. When indicated, diagnostic or therapeutic surgical procedures were performed. The indication for surgery was based on the results of the clinical examination; preoperative biomarker levels; the ultrasound findings, evaluated with the IOTA simple rules, as described by Timmerman et al.^(7)^; and the MRI results (practitioners did not have access to MRI scoring results). Figure 1 shows the patient selection process. Of the 257 women initially enrolled, 14 were lost to follow-up. Therefore, data for 243 women, with a total of 287 adnexal masses, were included in the study. Of the 169 patients (with 190 adnexal masses) for whom a histological diagnosis was made, 62 were found to have a single malignant adnexal tumor, 14 were found to have bilateral malignant adnexal tumors, 86 were found to have a single benign adnexal tumor, and 7 were found to have bilateral benign adnexal tumors. A team of pathologists specializing in pelvic neoplasms made the final histological diagnosis, in accordance with the guidelines established by the WHO Classification of Tumours of Female Reproductive Organs^(21)^. Of the 74 patients (with 97 adnexal masses) who did not undergo an invasive procedure and were followed to evaluate their clinical evolution, one had four adnexal masses, two had three adnexal masses, 16 had two adnexal masses, and 55 had a single adnexal mass. The last follow-up evaluation was in December 2020.

The MRI scans, surgical procedures, and histopathological analyses were performed at the Hospital da Mulher Prof. Dr. J. A. Pinotti. More than one tumor was found in 40 women, and the O-RADS MRI score was calculated for each mass separately, as suggested by its authors^(17)^. All MRI assessments were performed before the histological diagnosis was known or the decision for clinical follow-up had been made.

The reference standard was the histopathological diagnosis in the adnexal masses submitted to surgery (n = 178) and in those submitted to percutaneous biopsy (n = 12). For the adnexal masses not subjected to histopathological examination (n = 97), the criteria for benign disease were based on clinical and imaging data obtained over a period of at least 12 months, following the usual clinical care protocols of the institution.

### MRI

All MRI scans were acquired in a 1.5-T scanner (Signa HDxt; GE Healthcare, Milwaukee, WI, USA) with a pelvic phased-array coil. Prior the MRI scans, patients fasted for 3 h. No antispasmodic agents were used; nor was vaginal or rectal contrast administered. We used a protocol aimed at assessing adnexal masses, which consisted of T2-weighted multiplanar (axial, sagittal, and coronal) sequences, in-phase and out-of-phase T1-weighted sequences, diffusion-weighted sequences (*b* = 0, 500, and 1,000 s/mm^2^), and T1-weighted sequences, with and without fat saturation, before and after intravenous contrast administration by power injection at 3.5 mL/sec. The DCE study consisted of five sequential acquisitions, with an interval of 30 s between them and acquisition times ranging from 10 s to 13 s. The first sequence began 21 s after the intravenous injection of contrast. An additional upper abdomen diffusion-weighted sequence was performed in order to identify distant metastases (to solid organs or lymph nodes).

The MRI scans were analyzed by two radiologists, one specializing in MRI of the pelvis and the other specializing in MRI of the upper abdomen, who were working separately and were blinded to the histological diagnosis, as well as to the follow-up data. Both radiologists had vast prior experience (10 and 9 years, respectively) in the analysis of pelvic MRI scan. For all of the adnexal masses, each radiologist calculated the O-RADS MRI score independently. Disagreements regarding the final classification were resolved by consensus.

### O-RADS MRI score

Adnexal masses were described with terms established in the literature and endorsed by the American College of Radiology^(14,19)^, involving morphological features on MRI and the DCE standards necessary for applying the O-RADS MRI score. Table 1 illustrates the categories and main findings of the O-RADS MRI score.

**Table 1:** O-RADS MRI scoring system.

Score-risk category	PPV	MRI findings
1 - Normal ovaries	—	Non-ovarian lesion. Follicle (simple cyst ≤ 3 cm), corpus luteum, or hemorrhagic cyst in a premenopausal woman. Unilocular cyst with any type of fluid content (no enhancing wall or solid tissue*).
2 - Almost certainly benign	< 0.5%	Unilocular cyst with simple or endometrial fluid content (smooth enhancing wall and no enhancing solid tissue). Lesion with lipid content^†^ and no enhancing solid tissue. Lesion with “dark T2/dark DWI” solid tissue (homogeneously hypointense on T2 and DWI).
3 - Low risk	≈ 5%	Unilocular cyst with proteinaceous, hemorrhagic or mucinous fluid content (smooth enhancing wall and no enhancing solid tissue). Multilocular cyst with any type of fluid content and no lipid content (smooth septa, wall enhancement, and no enhancing solid tissue). Lesion with solid tissue (excluding T2 dark/DWI dark): low-risk (type 1) time-intensity curve on DCE MRI.
4 - Intermediate risk	≈ 50%	Lesion with solid tissue (excluding T2 dark/DWI dark): intermediate-risk (type 2) time-intensity curve on DCE MRI; if DCE MRI is not feasible, score 4 is any lesion with solid tissue (excluding T2 dark/DWI dark) that is enhancing ≤ myometrium at 30-40s on non-DCE MRI. Lesion with lipid content with large volume enhancing solid tissue.
5 - High risk	≈ 90%	Lesion with solid tissue (excluding T2 dark/DWI dark): high risk time-intensity curve on DCE MRI if DCE MRI is not feasible, score 5 is any lesion with solid tissue (excluding T2 dark/DWI dark) that is enhancing > myometrium at 30-40s on non-DCE MRI. Peritoneal, mesenteric, or omental nodularity or irregular thickening, with or without ascites.

PPV, positive predictive value (for malignancy); DWI, diffusion-weighted imaging.

* Solid tissue is defined as a lesion component that enhances and conforms to one of these morphologies: papillary projection; mural nodule; irregular septation/wall; or other larger solid portions.

† Minimal enhancement of Rokitansky nodules in a lipid-containing lesion does not change the classification to O-RADS MRI 4.

Sources: Thomassin-Naggara et al.^(17)^ and the American College of Radiology^(18,19)^.

### Statistical analysis

Data were analyzed using the R Environment for Statistical Computing Software^(22)^. We calculated the sensitivity, specificity, positive predictive value, and negative predictive value for O-RADS MRI scores, using ≥ 4 as the cutoff score for malignancy^(17,18)^. For statistical purposes, borderline ovarian tumors were classified as malignant.

## RESULTS

**Figure 2:**
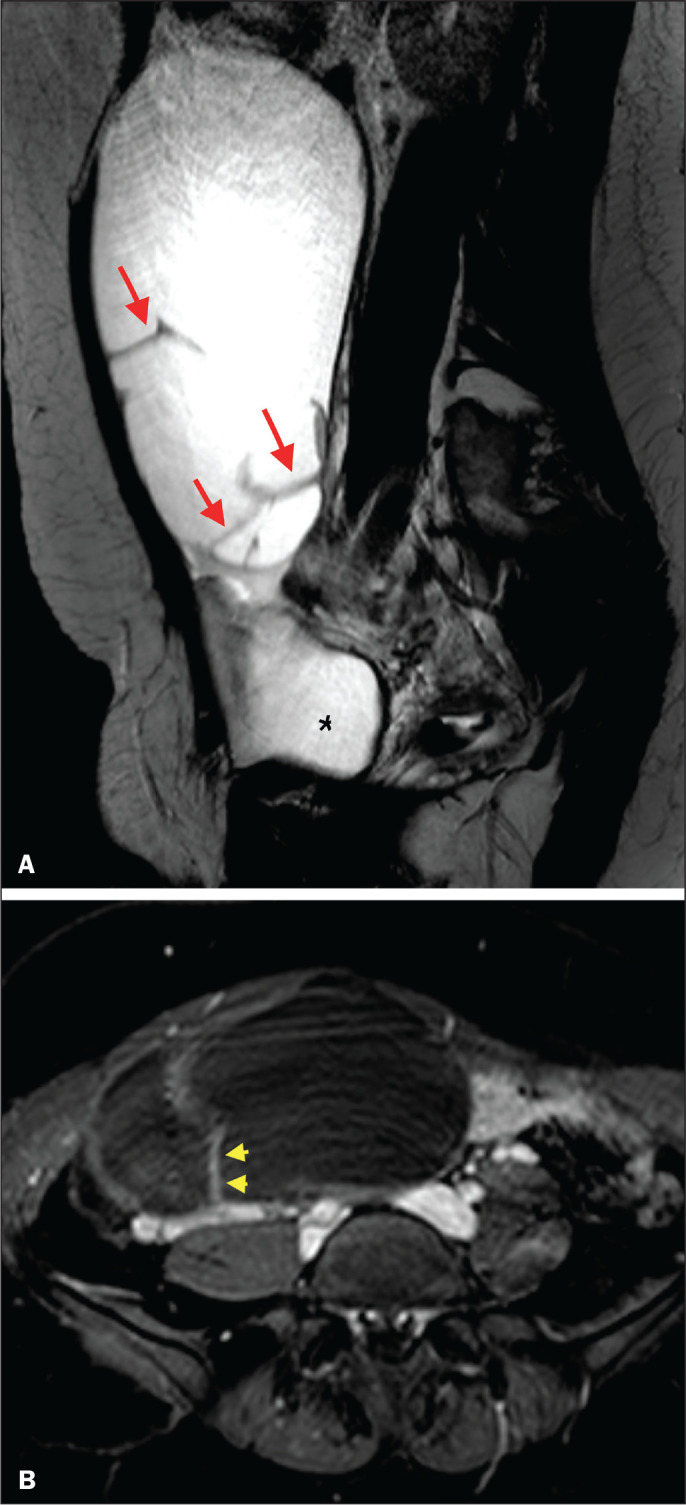
A 43-year-old woman with chronic pelvic pain and a right adnexal mass identified on ultrasound, with an indeterminate result based on the IOTA simple rules. A: T2-weighted sagittal sequence showing a cystic adnexal mass with multiple septa (red arrows) centered in the right adnexal region, near the bladder (asterisk). B: Contrast-enhanced axial fat-saturated T1-weighted sequence (acquisition at 30 s after contrast administration) showing enhancement of some septa (yellow arrowheads), with no solid portions. The final O-RADS MRI score was 3 (low risk). The patient underwent surgery (right oophorectomy), and the final histological diagnosis was mucinous cystadenoma.

The histological subtypes of benign and malignant adnexal masses are shown in Table 2. A final histopathological diagnosis was made in 190 (66.20%) of the 287 masses evaluated in the present study. Because our hospital is a tertiary cancer center, the malignancy rate was high, 90 (47.37%) of those 190 masses being classified as malignant in the histopathological analysis. Of the remaining 97 masses, which were followed clinically, none showed signs of malignant transformation, maintaining an O-RADS MRI score of 2 (almost certainly benign) or 3 (low risk). Figure 2 illustrates an adnexal mass in the right ovary, with an O-RADS MRI score of 3 (low risk), which was resected surgically. In that case, the final histopathological diagnosis was benign mucinous cystadenoma.

**Table 2:** Characteristics and final diagnoses of adnexal masses (n = 287).

Characteristic	n (%)
Means of establishing the diagnosis	
Imaging follow-up findings (≥ 1 year)	97 (33.80)
Histopathological results	190 (66.20)
Benign disease	100 (34.84)
Ovarian	
Endometrioma	1 (0.35)
Benign germ cell tumor	20 (6.97)
Cystadenoma	39 (13.59)
Stromal tumor	8 (2.79)
Endometrioma/endometriosis	11 (3.83)
Ovarian torsion or necrosis	3 (1.04)
Functional non-neoplastic cysts	7 (2.44)
Non-ovarian	11 (3.83)
Malignant disease	90 (31.36)
Ovarian borderline	17 (5.92)
Serous tumor	11 (3.83)
Mucinous tumor	4 (1.39)
Seromucinous tumor	2 (0.70)
Invasive malignant	73 (25.44)
Ovarian cystadenocarcinoma	44 (15.33)
Ovarian stromal tumors	5 (1.74)
Ovarian germ cell tumors	3 (1.04)
Anaplastic tumor	1 (0.35)
Metastasis	7 (2.44)
Non-ovarian tumor	13 (4.53)

Table 3 shows the final O-RADS MRI score for all adnexal masses, together with the sensitivity, specificity, positive predictive value, negative predictive value, positive likelihood ratio, and negative likelihood ratio, with cutoff O-RADS MRI scores of 4 and 5 for malignancy. The O-RADS MRI score showed a sensitivity and specificity of 91.11% and 94.92%, respectively, with an overall accuracy of 93.73%.

**Table 3:** Diagnostic performance of the O-RADS MRI score in adnexal masses (n = 287).*

Statistic^†^	Value	95% CI
Sensitivity	91.11%	83.23-96.08
Specificity	94.92%	90.86-97.54
Positive likelihood ratio	17.95	9.78-32.94
Negative likelihood ratio	0.09	0.05-0.18
Disease prevalence	31.36%	26.03-37.07
Positive predictive value	89.13%	81.71-93.77
Negative predictive value	95.90%	92.34-97.84
Accuracy	93.73%	90.27-96.24

* True-positive results = 87; false-positive results = 10; true-negative results = 188; false-negative results = 8.

† The sensitivity, specificity, positive predictive value, and negative predictive value were computed for dichotomized scores: scores of 1, 2, and 3 (benign) vs. scores of 4 and 5 (malignant).

Table 4 shows the adnexal masses for which the O-RADS MRI score produced a false-positive or false-negative result, together with the key imaging findings responsible for the diagnostic error. Among the eight cases of false-negative results, there were five malignant masses that did not present an identifiable solid portion, which resulted in an O-RADS MRI score of 2 or 3, and three malignant masses with a solid component that had a low-risk (type 1) time-intensity curve, which resulted in an O-RADS MRI score of 3. Among the ten cases of false-positive results, there was a moderate-risk (type 2) time-intensity curve, resulting in an O-RADS MRI score of 4, in all of the masses. None of the masses presented a high-risk (type 3) time-intensity curve.

**Table 4:** Details of adnexal masses erroneously categorized with the O-RADS MRI score (false positives and false negatives).

Result	Key imaging finding
False negative (n = 8)	
Five borderline tumors	No clearly solid component in four and a type 1 time-intensity curve in one
One mucinous ovarian carcinoma	No clearly solid component
One malignant Brenner tumor	Type 1 time-intensity curve
One endometrioid ovarian carcinoma	Type 1 time-intensity curve
False positive (n = 10)	
Four serous cystadenomas	Type 2 time-intensity curves
Three fibromas	Type 2 time-intensity curves
One thecoma	Type 2 time-intensity curve
One round ligament myoma	Type 2 time-intensity curve
One peritoneal inclusion cyst	Type 2 time-intensity curve

**Figure 3:**
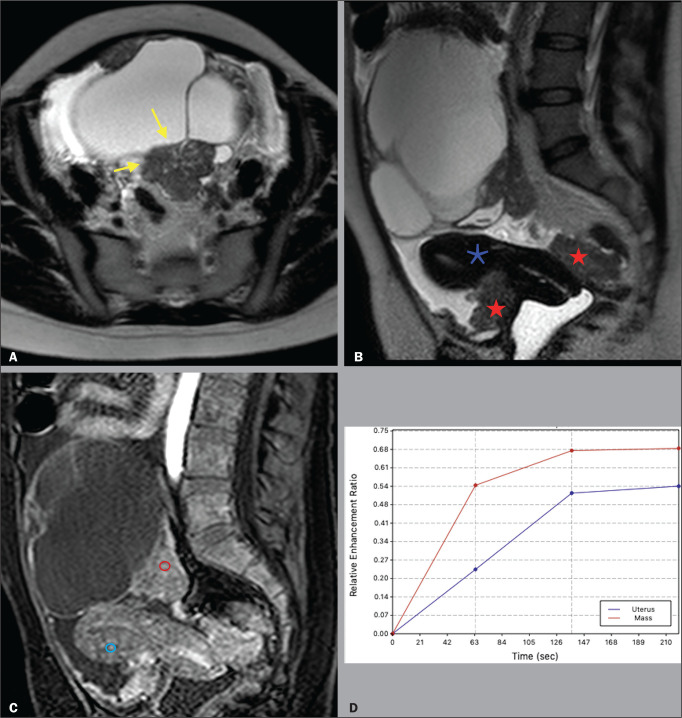
A 39 year-old woman with a family history of breast and ovarian cancer who presented with pelvic pain and a left adnexal mass. A: Axial T2-weighted sequence showing a multilocular cystic mass with solid portions (yellow arrows) centered in the left adnexal region. B: Axial T2-weighted sequence showing the multilocular cystic mass and multiple peritoneal implants (red stars) near the uterus (blue asterisk). C: Contrast-enhanced sagittal fat-saturated T1-weighted sequence (DCE study) showing visible enhancement of the solid component of the mass, greater than that of the myometrium, at 35 s after contrast administration. Note the region of interest over the uterus (blue circle) and the other over the adnexal mass (red circle). D: Relative enhancement ratio curve showing that the initial increase in the enhancement of the mass was greater than was that of the uterus. The final O-RADS MRI score was 5 (high risk). The patient underwent surgery, and the final histological diagnosis was high-grade serous cystadenocarcinoma with peritoneal carcinomatosis.

**Figure 4:**
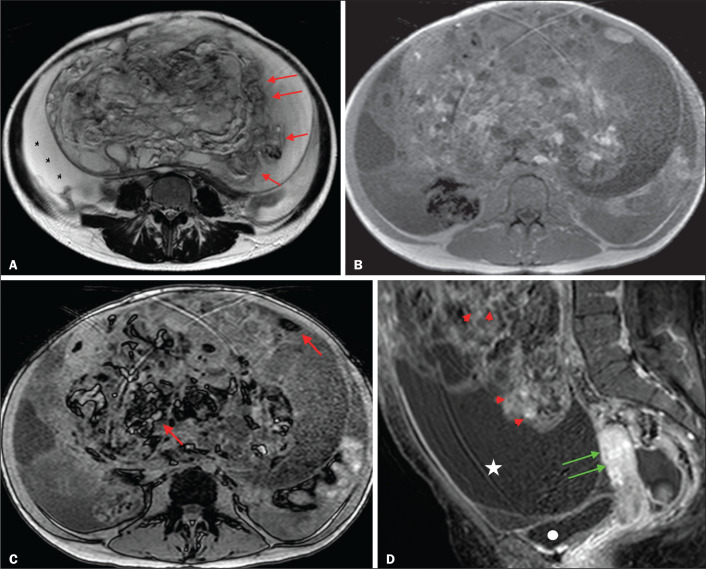
A 28-year-old woman with a left adnexal mass, extending to the upper abdomen, which had been considered indeterminate on ultrasound with the application of the IOTA simple rules. A: Axial T2-weighted sequence showing a solid-cystic mass (red arrows) centered in the left adnexal region. Note the ascites (asterisk). B,C: In-phase and out-of-phase T1-weighted sequences showing multiple foci of fat (signal drop in the out-of-phase sequence; red arrows), consistent with a germ cell tumor. D: Contrast-enhanced sagittal fat-saturated T1-weighted sequence (acquisition at 35 s after contrast administration) showing enhancement of the solid portions of the mass (red arrowheads) less than that of the myometrium (green arrows). Note the ascites (white star) and the location of the bladder (white circle). The final O-RADS MRI score was 4 (intermediate risk). The patient underwent surgery, and the final histological diagnosis was immature teratoma accompanied by gliomatosis peritonei.

Figure 3 illustrates an adnexal mass in the left ovary, showing the visible enhancement pattern at 35 s after injection of the contrast and the high-risk (type 3) time-intensity curve. For that mass, the O-RADS MRI score was 5 and the final histological diagnosis was high-grade serous cystadenocarcinoma with peritoneal carcinomatosis. Figure 4 shows a solid-cystic mass, centered in the left adnexal region and extending to the upper abdomen, with lipid content and a large volume of enhancing solid tissue. The O-RADS MRI score for such masses is 4^(18)^, and the final histological diagnosis in that case was immature teratoma accompanied by gliomatosis peritonei.

## DISCUSSION

The sensitivity, specificity, and overall accuracy of the O-RADS MRI score in the present study were similar to those reported for the original score^(17)^, which further supports its use in the assessment of adnexal masses, especially those considered indeterminate on ultrasound. In the O-RADS MRI protocol, it is necessary to include sequences aimed at evaluating the morphology of the adnexal mass—at least two T2-weighted multiplanar sequences (axial and sagittal or coronal) and T1-weighted sequences, with and without fat saturation, in order to stratify fat and blood components—and advanced sequences—diffusion-weighted sequences with *b* values of 800-1200 s/mm^2^ (enough *b* to suppress the T2 shine-through effect, thus ensuring that the urine in the bladder is black) and a DCE study, with a time resolution ≤ 15 s and a total time after gadolinium injection of 180 s. If DCE is unavailable (because of limitations of the magnet or software), the contrast uptake can be analyzed visually (at 30-40 s after gadolinium injection). In addition, the use of gadolinium can be foregone if no suspicious adnexal lesion is identified in the analysis of the conventional (T1- and T2-weighted) sequences, such as when only follicles or the corpus luteum are identified in a premenopausal patient.

The contrast uptake (DCE) study is the cornerstone of the O-RADS MRI score, defining the cutoff scores of 4 and 5. A moderate or marked increase in the signal intensity of an ovarian mass, in relation to that of the uterus, after gadolinium injection is associated with borderline and malignant tumors, correlating directly with the angiogenic status of a tumor^(23-25)^. In the initial study of the ADNEX MR SCORING system^(14)^, the DCE study images were obtained sequentially at intervals of 2.4 s, starting from 10 s after injection of the contrast, over a total of 320 s, with consequent post-processing on a workstation. That technical requirement was very rigid and complex, which could limit its use in clinical practice, a difficulty acknowledged by the authors^(15)^. The O-RADS MRI score allows the use of lower temporal resolution in the DCE study, with intervals of ≤ 15 s and a total acquisition time of 180 s, as well as including the option of performing a comparative visual analysis between the pattern of enhancement of the adnexal mass and that of the myometrium (at 30-40 s after gadolinium injection) when a DCE study is not available^(18)^. Those changes will allow the dissemination of the O-RADS MRI score to a greater number of MRI diagnostic centers, even those using low-field scanners, which is still a reality in low- and middle-income countries^(26)^.

Another update was the classification of adnexal masses with lipid content and a large volume of enhancing solid tissue as deserving of an O-RADS MRI score of 4^(18)^. That is important because some malignant tumors with a fat component, such as immature teratomas, can mimic benign lesions and need to be carefully evaluated^(27)^. However, minimal enhancement of Rokitansky nodules in a lesion containing lipid does not change the classification to an O-RADS MRI score of 4^(18)^. Therefore, according to the O-RADS MRI scoring system, not every adnexal mass with fat content is benign, especially in women under 20 years of age, with or without changes in serum biomarkers, lactic dehydrogenase, and alpha-fetoprotein^(28,29)^.

Certain diagnostic challenges persist, especially in cases of borderline tumors or invasive malignant tumors with type 1 time-intensity curves or without clearly solid portions, which were the main causes of the false-negative O-RADS MRI score results in the present study. The use of new diagnostic parameters and imaging concepts, especially radiogenomics studies^(30)^ and tailored imaging protocols for borderline malignancy^(31)^, could facilitate the stratification of such tumors.

Our study has some limitations. First, it was conducted at a tertiary cancer center, which increased the positive predictive value for malignancy in our sample. In addition, MRI reporting was performed by experienced examiners, whereas it is recommended that the reporting be performed by less experienced examiners or generalist radiologists. Furthermore, we did not assess the accuracy of the use of a single analysis of enhancement at 30-40 s after gadolinium injection in determining O-RADS MRI scores of 4 and 5, which could have altered our findings. Other authors have even evaluated the use of unenhanced images to construct the O-RADS MRI score, especially when clinical conditions, such as nephropathy or severe allergy^(32)^, preclude the use of contrast.

In summary, our data support the use of the O-RADS MRI score to assess adnexal masses, especially those considered indeterminate on ultrasound. The latest updates to the O-RADS MRI score facilitate its interpretation and will allow its use to become more widespread, with no loss of diagnostic accuracy.
